# Book Review: The Influential Mind: What the Brain Reveals About Our Power to Change Others

**DOI:** 10.3389/fpsyg.2019.01210

**Published:** 2019-05-22

**Authors:** Shubham Pandey, Rashmi Gupta

**Affiliations:** Cognitive and Behavioral Neuroscience Laboratory, Department of Humanities and Social Sciences, Indian Institute of Technology Bombay, Mumbai, India

**Keywords:** influential mind, persuasion, emotions, mental state, positivity

A few days back, I (the first author) had a heated discussion with my friend who believes that Psychology is not a science. Despite my presentation of multiple facts, findings, and arguments, I failed to convince her. Humans have successfully eradicated smallpox, landed on Mars, build jet engines, and complex internet. All it has been the result of cooperation, rational, and objective thinking. On the other hand, from politics to general discourse, people show irrational behavior, often fueled by prejudice, biases, gut feelings, conspiracy theories, etc. Why do people deny facts and scientific findings? Searching for the answer; I decided to read Tali Sharot's book: “The Influential Mind.”

Tali Sharot is a professor of cognitive neuroscience at the University College London where she runs “Affective Brain Lab,” investigating the neuroscience of motivation and emotion. Her previous book “The Optimism Bias” explores the basis of excessive positive thinking in humans (Sharot, 2011). Her new book “The Influential Mind” aims to explain the phenomenon of influence (changing others' mind) from three different perspectives (a) why we often fail to influence others, (b) what we can learn to influence others, and (c) how to recognize when others influence us. Sharot emphasizes that the only way to change others' mind is to align ourselves with them over seven core elements that regulate a person's' thoughts and actions. These seven core elements are prior beliefs, emotions, incentives, sense of agency, curiosity, state of mind, and the knowledge and acts of other people.

The first core element “prior” (our beliefs) is the key to effective communication. Because we do not want to change our beliefs easily, we take interest mostly in those communications which are in agreement with those beliefs. Further, if disagreement occurs, we tend to counter-argue and try to support our belief with new data from sources like the internet. Sharot argues that only offering facts and figures supporting our view and showcasing errors in other's arguments is not the best strategy. Instead, she suggests that building common grounds is necessary to influence others in any communication.

Since facts alone do not change people's mind, Sharot argues that people are influenced instantly, regularly, and unconsciously by other's “emotions,” and this is the second core element. Sharot emphasizes that positively framing our views is more powerful compared to negative. But why? It has been shown that less attentional resources are required to process positive information than negative information (Srivastava and Srinivasan, 2010; Gupta et al., 2016). Also, positive emotion broadens (while negative emotion narrows) our thought and actions (Fredrickson, 2004, for a review), while negative emotions might elicit inaction and demotivate others.

Complementing the above view, Sharot reemphasizes the power of positive emotions specifically rewards or “incentives,” which is the third core element. She suggests that immediate positive reward works better than a threat at a later time. Interestingly, the author hints that when the goal is to make someone not to do something, warning of adverse consequences may be more effective than promising rewards.

Humans tend to exercise control over others. The sense of control or “agency” is the fourth core element of Sharot's book. The author strongly points out that eliminating or reducing the sense of control of others leads to anger, frustration, and resistance thereby reducing the chances of any influence. On the other hand, enhancing people's sense of control over the choices being presented makes them more content, motivated, and compliant. In our view, it further builds trust and a sense of responsibility.

We all have a fundamental instinct: “Curiosity” (the desire to know), which is the fifth core element. We tend to seek out information that brings us hope and avoid information that brings us despair. Sharot points out that before transferring information to others, first, we need to highlight the existing gap in their knowledge and make them believe how knowing this information will make their world better.

To influence someone, we also need to pay attention to the current “mental/emotional state” of that person, which is Sharot's sixth core element. For example, in a stressful/threatening situation (e.g., a lion approaching toward you), we utilize all mental resources to survive (Mendl, 1999), leaving no resources to process the message given by others, hence, eliminating any chance of influence. Sharot emphasizes that the best strategy to influence someone is when the other person is calm or relaxed.

Finally, Sharot argues that many of our opinions and choices like the type of music, technology, names, etc. are influenced by the preferences of those around us, leading to group conformity (p. 140). However, the views (and acts) of majority may go wrong where the authenticity of the information can't be verified (e.g., social media, political campaigns), and hence, Sharot cautions us from being influenced in those situations.

Overall, this book can be seen as an extension of previous work on the Psychology of influence. For example, Gorman and Gorman (2016) have illustrated how people ignore facts and scientific findings due to prior beliefs and information gap, but that book was written primarily from a clinical perspective. Hence, someone who has previously read similar books or studied cognitive biases might find a few concepts and arguments redundant. However, the book is informative, entertaining and engaging, with practical points.

In conclusion, the book attempts to answer why it is difficult to change the attitude/s and actions of others. However, Sharot emphasizes the power of positive emotions. Using a positive approach we can influence others efficiently because we are biased to move toward rewards and away from pain or punishment. In emotion science research, mostly the study of positive emotions has been neglected, and an emphasis is given mainly to negative emotions (Gupta, 2019). Many recent studies including Sharot's book shows the significance of positive emotions in our daily life. It not only influences our behaviors, belief, decision making but also optimizes our health and well being. The current need is to pay equal emphasis to study positive and negative emotions.

## Author Contributions

All authors listed have made a substantial, direct and intellectual contribution to the work, and approved it for publication.

### Conflict of Interest Statement

The authors declare that the research was conducted in the absence of any commercial or financial relationships that could be construed as a potential conflict of interest.
